# Vulnerability of peri‑urban and residential areas to landscape fires in Greece: Evidence by wildland-urban interface data

**DOI:** 10.1016/j.dib.2020.106025

**Published:** 2020-07-14

**Authors:** Ioannis Mitsopoulos, Giorgos Mallinis, Alexandros Dimitrakopoulos, Gavriil Xanthopoulos, Giorgos Eftychidis, Johann Georg Goldammer

**Affiliations:** a Directorate of Biodiversity and Natural Environment Management, Ministry of Environment and Energy, Patission 147, 11251 Athens, Greece; b Laboratory of Forest Remote Sensing, Department of Forestry and Management of the Environment and Natural Resources, Democritus University of Thrace, 68200 Orestiada, Greece; c Laboratory of Forest Protection, Department of Forestry and Natural Environment, Aristotle University of Thessaloniki, 54636, Thessaloniki, Greece; d Institute of Mediterranean Forest Ecosystems, Hellenic Agricultural Organization "Demeter", Terma Alkmanos, 11528, Athens, Greece; e Center for Security Studies, P. Kanellopoulou 4, 101 77, Athens, Greece; f Global Fire Monitoring Center (GFMC), Max Planck Institute for Chemistry, Georges-Koehler-Allee 75, d-79110 Freiburg, Germany

**Keywords:** Fire management, Spatial analysis, Burnt areas, Multitemporal changes, WUI, GIS, Geographic information system

## Abstract

We provide a map of wildland urban interface (WUI) areas at national scale in Greece, using as primary data the Corine Land Cover (CLC) 2018 product. The WUI areas distribution in Greece is calculated for all the regional units of the county. The regional units correspond to NUTS (Nomenclature of Territorial Units for Statistics) level 2 for Greece, being important for the national to regional fire risk management and mitigation within the country. Furthermore, spatially explicit information regarding the fire affected areas and the changes in WUI areas between 2006 and 2018 on the basis of the CLC data for each regional unit is also provided.

This geospatial information can provide valuable, concurrent information at no-cost to all responsible and competent authorities involved in landscape fire management in Greece and represent a valuable contribution to assist in national and regional scale planning.

Specifications Table

**Table:** 

Subject	Management, Monitoring, Policy and Law
Specific subject area	Landscape fire management at national and regional level
Type of data	Image Graph
How data were acquired	The raw data are extracted from different sources and analysed by a GIS software (ArcGIS) analysis. We assessed the fire hazard in Greece related to WUI surface area
Data format	Maps and Graphs
Parameters for data collection	Parameters were identified after a review of existing EU approaches and considering existing information gaps within the Greek state. We considered appropriate scale of the analysis and use of open-source data. EU and national repositories containing the necessary data were identified.
Description of data collection	The maps depict WUI areas, burned areas and respective burned surface within WUI area of the Greek regional units
Data source location	Corine Land Cover (CLC) 2018https://land.copernicus.eu/pan-european/corine-land-cover/clc2018 Corine Land Cover (CLC) 2006 https://land.copernicus.eu/pan-european/corine-land-cover/clc-2006 Regional Units of Greece https://www.statistics.gr/documents/20181/1194366/perif_enot.rar/1d3746c9–88df-4cfb-87fb-d8199f1f345b ESA's Climate Change Initiative (CCI) FireCCI51 burned area product https://www.esa-fire-cci.org/FireCCI51
Data accessibility	With the article
Related research article	Goldammer, JG, Xanthopoulos, G., Eftychidis, G., Mallinis, G., Mitsopoulos, I., Dimitrakopoulos, A., 2019. Report of the Independent Committee tasked to analyze the Underlying Causes and Explore the Perspectives for the Future Management of Landscape Fires in Greece, GFMC.

Value of the Data

• The wildland-urban interface (WUI) is the area where houses and other development meet or intermingle with undeveloped natural areas, resulting to pronounced wildfire risk. This information is essential for fire risk management and mitigation.
• The data can be used for the national assessment and identification of the increased fire hazard WUI areas from Greek forest and fire management authorities.
• This dataset can be used to prioritize fire risk control and develop better landscape fire risk management strategies, develop fire and fuel management plans and strategies to reduce the risk of costly and dangerous fires in WUI areas, plan wildfire mitigation activities. At policy level they can prioritize funding, develop or alter existing industrial fire mitigation regulations, and improve settlement, housing and infrastructure fire mitigation standards in WUI areas.
• A variety of fire decision-support activities may benefit from these data, including resource pre-positioning, values protection, evacuation planning and fire risk modeling. The national scale WUI maps can be used for prioritizing and implementing local scale, fire risk plans. These detailed maps can support the development of fire resilient communities.
• Currently, prioritization of high fire hazard areas in the country relies on a map included in the 575 Presidential Decree, issued in 1980, at a time when most of the current WUI areas did not yet exist. There is need to upgrade it to a national fire risk map including the impact of fire to WUI areas.
• The national scale WUI data can be also used to identify areas where detailed ecosystem services mapping and assessment are needed since they represent areas of intermix between people and natural vegetation, being crucial for biodiversity and ecosystem services monitoring.

## Data description

**Fig. 1:**
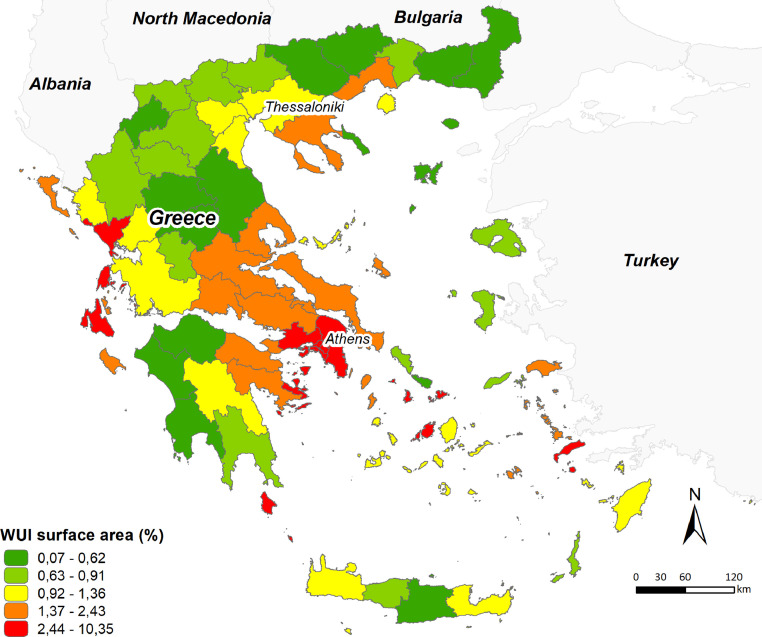
The wildland-urban interface (WUI) map of Greece at regional unit level derived from CLC 2018.

**Fig. 2:**
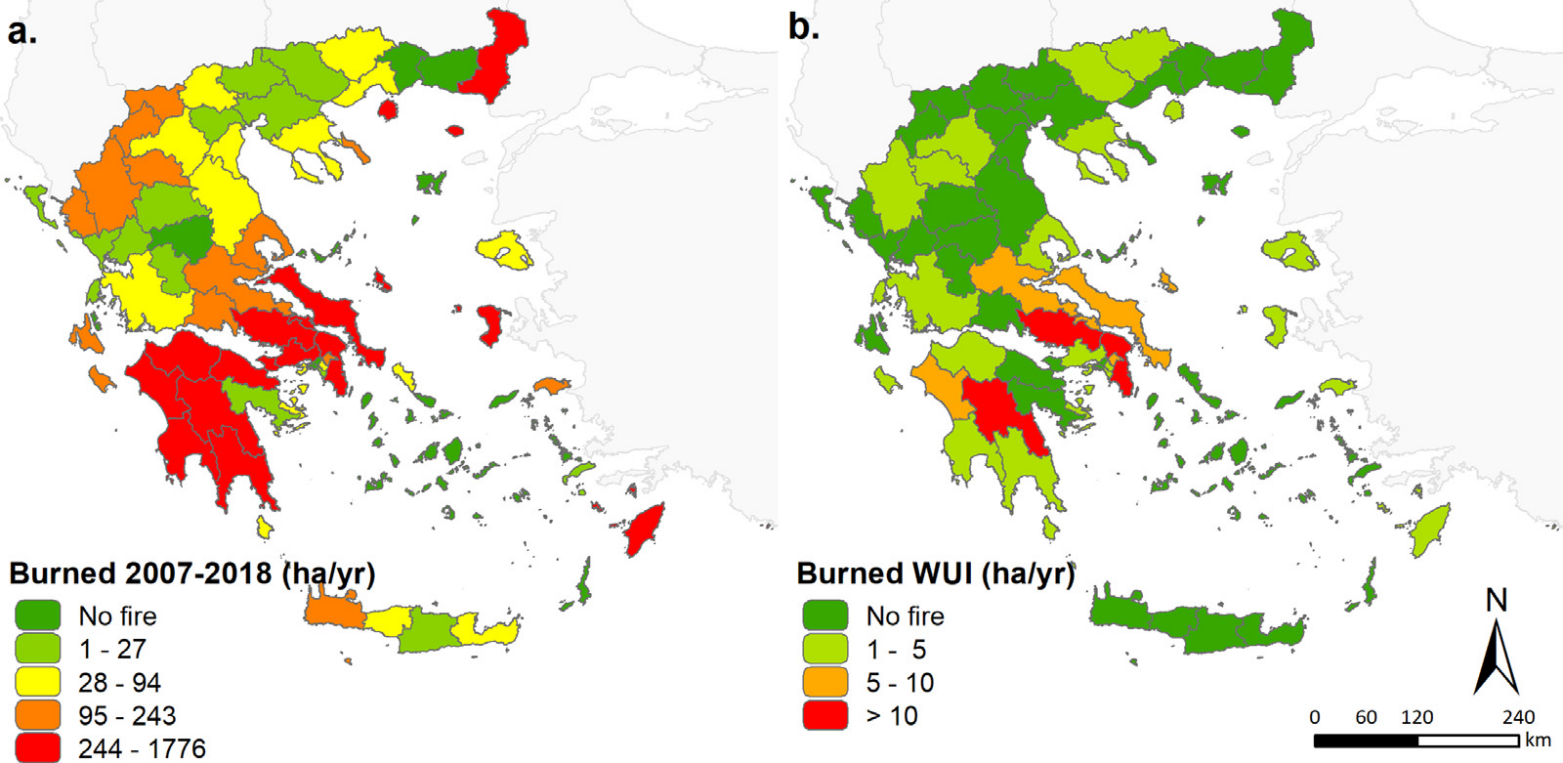
Burned area within each regional unit (a) and respective (b) during 2007–2018.

**Fig. 3:**
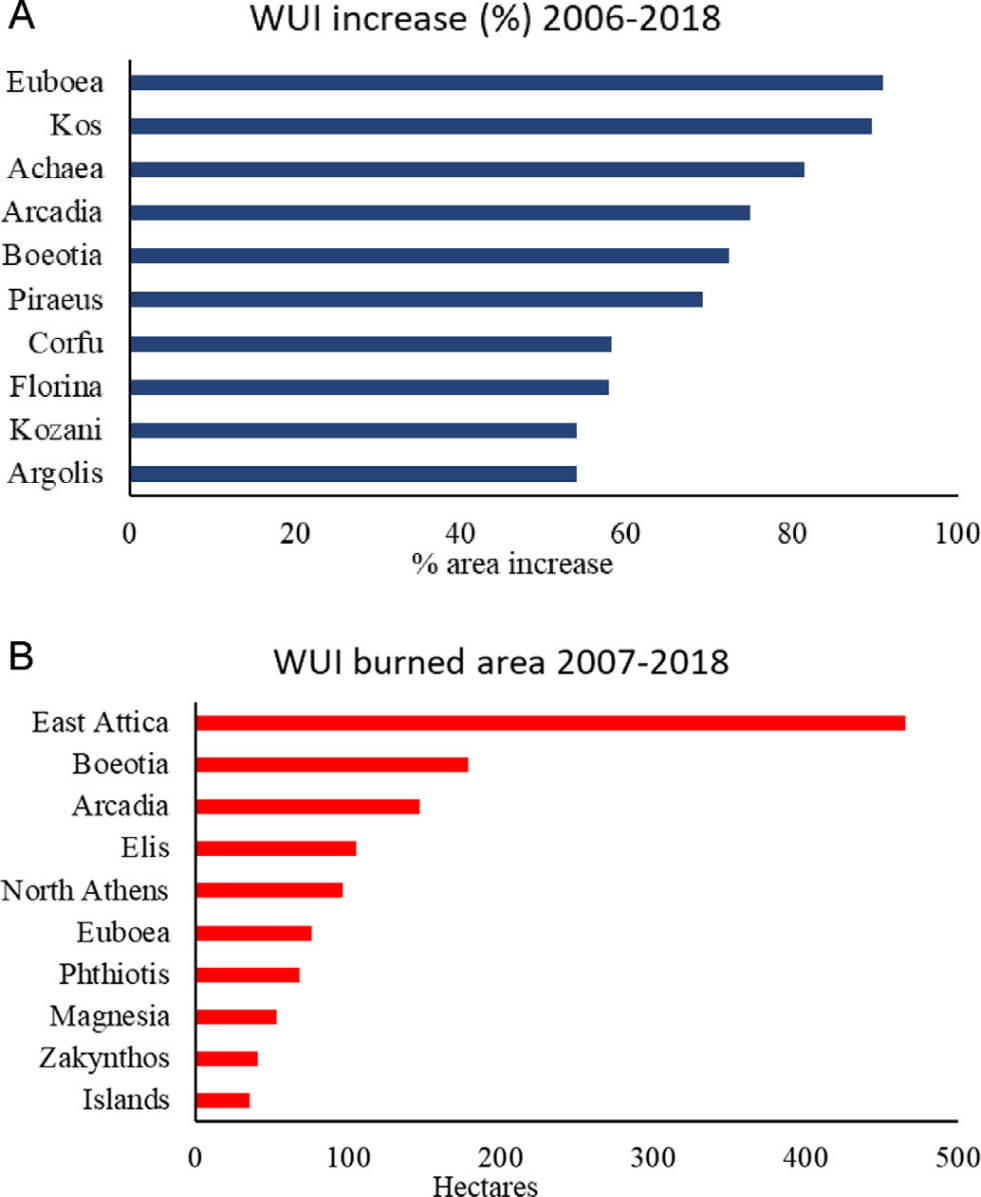
The ten regional units presenting the highest WUI area increase between 2006 and 2018 (left) and the ten regional units with the largest burned WUI areas (right).

The maps presented within the manuscript (Fig. 1, Fig. 2) were generated by the main data of our analysis, available as supplementary material in Appendix A (named as WUI_Regional_Units_of_Greece.shp), containing information regarding the burned area and respective burned surface within WUI areas of each regional unit in Greece during 2007–2018. Also, within the manuscript a graph is presented (Fig. 3), illustrating the ten regional units presenting the highest WUI area increase between 2006 and 2018 and the ten regional units with the largest burned WUI areas. This visualization is generated upon a tabular .csv data (named as WUI_Greece_change.csv) available as supplementary material in Appendix A. The attribute table of the main data in Appendix A (WUI_Regional_Units_of_Greece.shp) stores information for the burned area for each Regional Unit (cci_ha_ann), the burned surface within WUI areas, for each Regional Unit (wuiburn_an) and the wildland-urban interface (WUI) area, for each Regional Unit as a percentage of total Regional Unit.

Additional data are provided as supplementary material in Appendix B, including geospatial datasets in raster GeoTIFF format depicting wildland-urban interface (WUI_2006.tif, WUI_2018.tif), fuel areas (Fuel_2006.tif, Fuel 2018.tif), artificial areas (Artificial_2006.tif, Artificial_2018.tif) in Greece for 2006 and 2018.

The geospatial data for fuel (i.e. Fuel_2006.tif and Fuel 2018.tif) and artificial (i.e. Artificial_2006.tif and Artificial_2018.tif) areas in Greece, include an attribute field indicating the spatial distribution of the respective classes as well as the associated buffers generated according to the methodology adopted.

Fig. 1 shows the WUI map of Greece at regional unit level, where the regional clusters with high WUI fractions, and therefore with increased fire hazard, are highlighted . The Chalkidiki and the northeast Peloponnesus peninsulas, along with several regions in the eastern central Greece also show high WUI densities.

Statistics show that the largest forest areas burned occur in the Iliea, Arkadia and Messinia regional units (Fig. 2a). Yet, the highest WUI burned area is identified over the East Attica regional unit (465 ha) (Fig. 2b and Fig. 3), where the deadliest fire (i.e. Mati fire) in Greek forest fire records occurred. In recent years (2006–2018), a rapid expansion of WUI areas is observed in regional units with low levels of fire activity (Fig. 3).

## Experimental design, materials and methods

### Geospatial data

In order to delineate WUI areas at national scale, the CORINE Land Cover (CLC) inventory of land use/land cover was used as a primary source of information. The CORINE Land Cover (CLC) is a European program, coordinated by the European Environment Agency (EEA), providing consistent information on land cover and land cover changes across Europe. Corine uses a Minimum Mapping Unit (MMU) of 25 hectares (ha) for areal phenomena and a minimum width of 100 m for linear phenomena. In the majority of EU countries (as in Greece) delineation of the 44 classes included in the land cover inventory is accomplished through visual interpretation of satellite imagery. For the CLC 2018 version, Sentinel-2 and Landsat-8 medium spatial resolution images have been used with the thematic accuracy exceeding 85% [1]. While the MMU of the CLC inventories might hinder detection of fine scale landscape elements, an attractive feature of the dataset is the consistency in terms of the technical guidelines and the availability over five periods (1990, 2000, 2006, 2012 and 2018), facilitating change analysis studies. Furthermore, in Greece, currently there is no national cartographic product providing information with higher thematic and geometric accuracy than CLC regarding the distribution of the land cover types of interest (i.e. natural, semi-natural, impervious etc.).

The Burned Area (BA) product (Fire_cci BA version 5.1) developed as part of the ESA CCI Programme for the generation and provision of Essential Climate Variables (ECV) on global scale is based on long-term satellite data time series of Terra Moderate Resolution Imaging Spectroradiometer (MODIS) optical images. The Fire_cci BA products is generated at 250 m, upon the surface spectral reflectance of bands 1 and 2 at 250 m (MOD09GQ Collection 6), along with complementary information of the MODIS Thermal anomalies/fire locations (MCD14ML Collection 6) and ESA's Land Cover CCI [2]. The Fire_cci BA products are available to the public through the Fire_cci website https://esa-fire-cci.org/ for a time span of 15 years (2003–2018).

The administrative boundaries were downloaded from the Hellenic Statistical Authority website providing Digital Cartographical Data used during the various census implementations nationwide [3].

### Spatial analysis

The methodological workflow follows the approach of Modugno et al. [4] who developed WUI maps for EU (except for Greece) and non-EU member states. The spatial analysis implemented for WUI mapping at national scale for 2018, included as a first step the identification of the wildland fuels and artificial areas within the country from the selection of CLC level 3 classes. In order to ensure consistency with Modugno et al. [4], we characterized as artificial surfaces continuous urban fabric (CLC 111), discontinuous urban fabric (CLC 112), dump sites (CLC 132), industrial or commercial units (CLC 121), construction sites (CLC 133) and sport and leisure facilities (CLC 142). On the other hand, broad-leaved forest (CLC 311), coniferous forest and transitional woodland-shrub (CLC 324) were characterized as wildland fuels.

Subsequently a buffer zone was calculated both for wildland fuels (400 m) and artificial surfaces (200 m). Subsequently, the wildland fuel areas and their respective buffers falling within the artificial area buffer, were identified as WUI areas.

The tabulation of the Greek regional units with the 2018 WUI areas, provides the WUI relative distribution map of Greece at national scale. Using the quantile method of classification, the whole territory can be prioritized for actions and measures within the framework of a coherent national fire management policy [5], [6], [7].

Using a similar spatial overlay analysis, our cartographic data was populated with the WUI distribution for 2006, allowing integration at the EU level with the information provided by Modugno et al. [1] as well as identifying areas under urbanization pressure within the last 12 years in Greece. Likewise, with a spatial overlay procedure and the use of the Fire_cci BA product, we provide information on the burned area within each regional unit and the relative distribution of the fire affected WUI areas.

This information is complementary to the 2018 WUI map for efficient fire management development.

## Declaration of Competing Interest

The authors declare that they have no known competing financial interests or personal relationships which have, or could be perceived to have, influenced the work reported in this article.

## Appendix Supplementary materials

Image, application 1

Image, application 2

Image, application 3
